# Observation of phonon trapping in the continuum with topological charges

**DOI:** 10.1038/s41467-020-19091-3

**Published:** 2020-10-15

**Authors:** Hao Tong, Shengyan Liu, Mengdi Zhao, Kejie Fang

**Affiliations:** 1 Holonyak Micro and Nanotechnology Laboratory and Department of Electrical and Computer Engineering, University of Illinois at Urbana-Champaign, Urbana, IL 61801 USA; 2 Illinois Quantum Information Science and Technology Center, University of Illinois at Urbana-Champaign, Urbana, IL 61801 USA

**Keywords:** Photonic crystals, Applied physics, Optomechanics, Quantum mechanics

## Abstract

Phonon trapping has an immense impact in many areas of science and technology, from the antennas of interferometric gravitational wave detectors to chip-scale quantum micro- and nano-mechanical oscillators. It usually relies on the mechanical suspension—an approach, while isolating selected vibrational modes, leads to serious drawbacks for interrogation of the trapped phonons, including limited heat capacity and excess noises via measurements. To circumvent these constraints, we realize a paradigm of phonon trapping using mechanical bound states in the continuum (BICs) with topological features and conducted an in-depth characterization of the mechanical losses both at room and cryogenic temperatures. Our findings of mechanical BICs combining the microwave frequency and macroscopic size unveil a unique platform for realizing mechanical oscillators in both classical and quantum regimes. The paradigm of mechanical BICs might lead to unprecedented sensing modalities for applications such as rare-event searches and the exploration of the foundations of quantum mechanics in unreached parameter spaces.

## Abstract (web summary)

Typically, phonon trapping is performed using mechanically suspended structures which have many limitations. Here the authors study a phononic structure that supports mechanical bound states in the continuum (BICs) at microwave frequencies with topological features.

## Introduction

Phonon trapping in low-dissipative mechanical systems, such as pristine piezoelectric crystals^1^ and bulk/surface acoustic wave resonators^2^, has played a crucial role in widespread technologies, including timekeeping and microwave signal processing. In principle, trapping phonons, i.e., excitations of crystal lattice vibrations, can be achieved in detached structures, in contrast to photons which permeate even in the vacuum. Nonetheless, complete mechanical isolation from the environment is unfeasible with common practices, and trapping phonons with the ultimate lifetime is an outstanding challenge. Recently, phonon-loss mechanisms and means to enhance the mechanical coherence have attracted intensive studies driven by the incentive to scale macroscopic mechanical resonators to microscales and nanoscales^3^ for realizing, for example, ultrasensitive mass sensors^4,5^ and quantum mechanical oscillators^6–8^. However, while trapping phonons in isolated vibrational modes, suspended structures with reduced lateral dimensions cause poor thermalization and excess noises when mechanical oscillators are coupled to external probes. These issues are more pressing when coupling optical photons with the trapped phonons, precluding continuous measurements of quantum mechanical oscillators at cryogenic temperatures^9,10^ and preparation of high-fidelity quantum mechanical states in ambient conditions^11,12^.

Originally conceived in peculiar quantum mechanical potentials^13^, bound states in the continuum (BICs) are nonradiative states yet spectrally overlapping with the continuum, because of symmetry incompatibility with the radiative modes^14^ or accidental radiation amplitude cancellation^15^. Recently, both types of optical BICs have been observed in two-dimensional photonic crystal slabs^16–18^, rendering macroscopic thin-film optical resonators with a quality factor comparable to those of microcavities^19^.

**Fig. 1:**
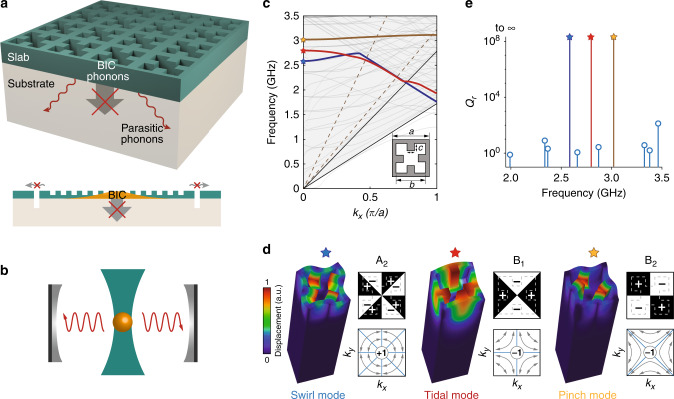
Phonon trapping via mechanical BICs. **a** Schematic diagram of a slab-on-substrate PnC which traps selected phonons via mechanical BICs while facilitates dissipation of parasitic phonons via the substrate. **b** The mechanical BIC architecture resembles levitated particles. **c** Calculated mechanical bandstructure of an AlN-on-oxide PnC with parameters given in the text. The solid (dashed) lines correspond to the sound lines of transverse and longitudinal waves of SiO_2_(AlN), respectively. Three mechanical BICs at the *Γ* point and the associated mechanical bands with the consistent symmetry are highlighted. **d** Simulated total displacement of the three BIC modes (swirl, tidal, and pinch) and their group representation under *C*_4v_ symmetry. Also shown is the far-field transverse polarization of Bloch modes of the same band in the vicinity of *Γ* point, identifying the topological charge of mechanical BICs. Blue lines are the nodal lines of far-field longitudinal polarization. a.u. arbitrary units. **e** Simulated radiative quality factor of mechanical modes at the *Γ* point.

Here, we show an approach for phonon trapping in a chip-scale architecture via mechanical BICs, which is distinctive to the method of mechanical bandgap engineering widely used in suspended structures^20–22^. We propose mechanical BICs realized in slab-on-substrate phononic crystals (PnCs), as illustrated in Fig. 1a, where phonons in the BIC mode are trapped in the unreleased slab without coupling into the substrate, irrespective of the acoustic impedance of the slab and substrate materials, while parasitic phonons are dissipated via the substrate. By further introducing voids on the boundary of the PnC, the radiation loss of BIC-phonons thus can be eliminated, resembling optically trapped nanoparticles (Fig. 1b), but without the need to levitate the structure, leading to unmatched heat capacity. As our experiments and simulations show, both symmetry-induced and accidental mechanical BICs with topological features can be realized in slab-on-substrate PnCs. In contrast to confined surface or slab acoustic waves with finite momentum below the sound line, mechanical BICs with zero wavevectors are able to couple with single optical resonances in two-dimensional periodic structures^16,17^, for noninvasive, optical interrogation of the trapped phonons, similar to the prevailing cavity-optomechanical approaches^23^.

## Results

### Room temperature spectroscopy of mechanical BICs

We designed two-dimensional PnCs which support mechanical BICs in the thin-film aluminum nitride (AlN)-on-oxide structure^24^—a material system which otherwise is free of confined microwave-frequency acoustic waves in a uniform slab because of the much larger Young’s modulus of AlN than SiO_2_. Figure 1c shows the mechanical bandstructure of a PnC with *C*_4v_ symmetry and unit-cell dimensions (*a*, *b*, *c*) = (1000, 800, 200) nm in a structure with 600 nm AlN and 4 μm SiO_2_ on silicon. We numerically found three symmetry-induced mechanical BICs at the *Γ* point, which decouple from both transverse and longitudinal acoustic waves in the substrate, with a frequency and group representation of (2.58 GHz, *A*_2_), (2.80 GHz, *B*_1_), and (3.02 GHz, *B*_2_), respectively, and their modal profile and radiative quality factor shown in Fig. 1d, e. Despite the fundamentally different nature of acoustic and electromagnetic waves, we found that mechanical BICs are associated with transverse topological charges, i.e., the winding number of the far-field transverse polarization of Bloch modes in the vicinity, similar to the optical counterparts^25^, and yet connected with longitudinal nodal lines in the Brillouin zone, where the far-field longitudinal polarization vanishes (Supplementary Note 1), as illustrated in Fig. 1d. In addition, we also find robust existence of accidental mechanical BICs with integer topological charges in slab-on-substrate PnCs despite *z*-symmetry breaking (Supplementary Note 1), in contrast to the optical case^26^, which can be attributed to the confinement of phonons in solids.

**Fig. 2:**
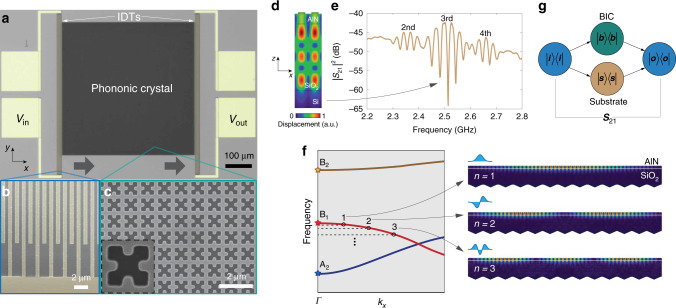
Piezoelectric detection of mechanical BICs. **a** False-color optical microscopy image of a device consisting of a PnC with 500 × 500 unit cells and a pair of IDTs with electrodes. **b**, **c** Scanning electron microscopy images of the IDT fingers (**b** false-color) and the PnC (**c**). **d** Simulated modal profile of the third-order AlN-SiO_2_ eigenmode. **e** Measured transmission spectrum via a pair of IDTs. **f** Formation of standing-wave resonances near the band edge in finite-size PnCs and the simulated modal profile of the first three band-edge modes formed from the *B*_1_ BIC in a *y*-periodic PnC. **g** Schematic diagram of the measurement protocol.

We fabricated the designed PnCs in AlN-on-oxide silicon microchips (see “Methods” section), together with interdigital transducers (IDTs) for piezoelectric actuation of the mechanical BICs (Fig. 2a–c). Because of the reflection at the oxide-silicon interface, the AlN–SiO_2_ stack supports weakly confined acoustic modes with energy distributed in the AlN slab^27^, which can be piezoelectrically excited by IDTs (Fig. 2d). We first measured devices without PnCs to characterize the IDT response, with Fig. 2e showing the measured transmission spectrum via a pair of IDTs separated by 100 μm, each of which has 20 pairs of fingers and a periodicity of 1.708 μm. The spectrum exhibits three major envelops whose center frequency corresponds to the second-order, third-order, and fourth-order AlN–SiO_2_ modes, while the fringes under the envelope are due to the reflection between the two IDTs forming a weak Fabry–Perot cavity. By tuning the periodicity of IDT fingers, we can adjust the frequency of the AlN–SiO_2_ modes for them to couple with selected mechanical BICs.

In actual PnCs with *N* × *N* unit cells, the continuous mechanical bands near the band edge discretize into standing-wave modes with constituent momenta  ±*k*_*x,y*_ defined relative to the band edge, approximately satisfying the resonance condition (*k*_*x*_, *k*_*y*_) = (*n*, *m*)*π*/Na, *n*, *m* ≥ 1, which we label as the (*n*, *m*)-th mode. For example, Fig. 2f shows the simulated modal profile of the first three (*n* = 1, 2, 3) band-edge modes near the *Γ* point of the mechanical band associated with the *B*_1_ BIC. As illustrated in Fig. 2g, upon intersecting with the PnC, IDT-excited AlN-SiO_2_ modes split, where the slab part couples with the BIC standing-wave modes and the rest propagates as a separate substrate mode, i.e., a standing wave mode in SiO_2_, resulting in the transmission coefficient *S*_21_ an averaged mixture of the two parts (see “Methods” section). Since the portion of acoustic energy in the slab is significantly less than that in the substrate for the AlN-SiO_2_ mode, the transmission spectrum will comprise weak BIC standing-wave resonances on top of IDT fringes.

**Fig. 3:**
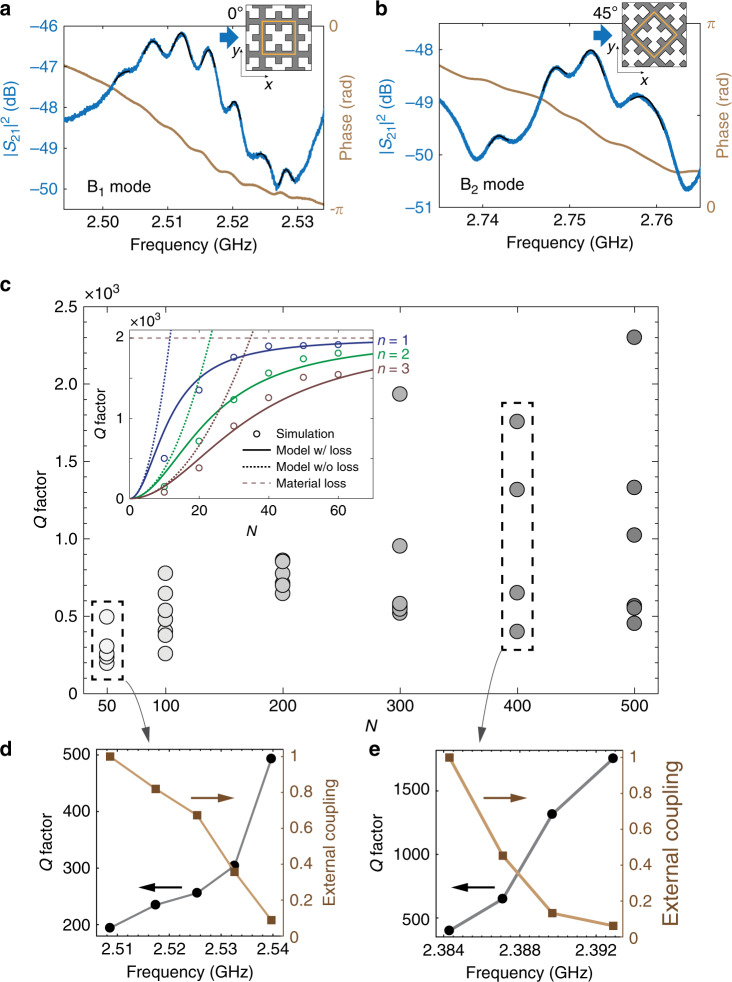
Room-temperature mechanical spectroscopy. **a**, **b** Measured transmission spectrum of a 0° (**a**)-oriented and 45° (**b**)-oriented PnC exhibiting *B*_1_ and *B*_2_ standing-wave modes, respectively. The black curves are the fitted transmission at resonances. For a broadband spectrum see Supplementary Note 3. **c** Observed room-temperature *Q* factors of *B*_1_ standing-wave resonances versus the size of PnCs with *N* × *N* unit cells. The inset shows the *Q* factor of the first three *B*_1_ standing-wave modes in *y*-periodic PnCs by numerical simulation (circles) and theoretical modeling. Material damping of 0.1% is introduced in the simulation. **d**, **e** *Q* factor and normalized external coupling (*ω*_m_/*Q*_e_, relative to the highest one) of the resonances in PnCs for *N* = 50 and 400.

We first made PnCs with the square unit cell aligned with the IDT fingers. Since the IDT-excited acoustic waves are even about the *x*-axis, i.e., the wave propagation direction, only the *B*_1_ BIC that is *x*-mirror even will be excited. Figure 3a shows the power transmission coefficient of a PnC with 100 × 100 unit cells measured at room temperature, where several standing-wave modes formed from the *B*_1_ BIC are observed in the frequency range of 2.5–2.53 GHz. The frequency discrepancy from the simulation can be attributed to material properties (e.g., the polycrystalline AlN) and fabrication imperfection. To probe the *B*_2_ BIC, we rotated the PnC by 45° so that the rotated *B*_2_ mode is *x*-mirror even and thus can be excited by the incident acoustic wave (Fig. 3b), while the *B*_1_ BIC becomes obscured in this setting. On the other hand, the *A*_2_ BIC is inaccessible in either case because of its oddness under both *x*-mirror and diagonal-mirror operations.

### Quality factor of mechanical BICs

As a new method for phonon trapping, we conducted a comprehensive study of the acoustic loss of mechanical BICs. For the mechanical BIC standing-wave mode, its dissipation can be attributed to three main sources: the radiation loss into the substrate and along the lateral direction, phonon scattering loss caused by the inhomogeneity of the fabricated PnC which breaks spatial periodicity, and material related losses^28,29^. In terms of the mechanical quality factor *Q*, i.e., the ratio between the frequency and loss rate, we have

1 $$\frac{1}{Q}\equiv \frac{1}{{Q}_{{{e}}}}+\frac{1}{{Q}_{{{i}}}}=\frac{1}{{Q}_{{{e}}}}+\frac{1}{{Q}_{{{r}}}}+\frac{1}{{Q}_{{{s}}}}+\frac{1}{{Q}_{{{a}}}},$$

where *Q*_e_ is the external quality factor related to the lateral radiation for probing the BICs, and *Q*_i_ is the intrinsic quality factor including components *Q*_*r,s,a*_, i.e., the substrate-radiation, phonon-scattering, and material-absorption limited quality factors, respectively. The radiative quality factor *Q*_r_ for the (*n*, *m*)-th BIC standing-wave mode in a *N* × *N* PnC can be modeled as (Supplementary Note 2)

2 $${Q}_{r}\propto {\left(\frac{{n}^{2}\,+\,{m}^{2}}{{N}^{2}}\,+\,\zeta {\alpha }^{2}\right)}^{-1},$$

where *α* is a parameter measuring the symmetry-breaking perturbation of the unit cell and *ζ* > 0 is a constant for a given BIC. Phonon scattering occurs between isofrequency modes, leading to increased radiation of a BIC standing-wave mode according to Fermi’s golden rule. For band-edge modes in the vicinity of the *Γ* point, we show that *Q*_*s*_ follows the same scaling rule as *Q*_*r*_ in sufficiently large PnCs (Supplementary Note 2). Finally, we assume that *Q*_*a*_ to be approximately device-size and mode independent at room temperature.

We fabricated a group of 0^∘^-oriented PnCs with different sizes and observed standing-wave resonances formed from the *B*_1_ BIC in each PnC. The fitted total quality factor of these resonances is summarized in Fig. 3c. For a given size of PnC, the resonances with higher frequency generally have larger *Q* factor and less external coupling (e.g., Fig. 3d, e), consistent with the fact that these are lower-order modes as the mechanical band associated with the *B*_1_ BIC bends downward near the *Γ* point (Fig. 2f). This is in contrast to the *B*_2_ BIC, whose associated band bends upward near the *Γ* point, leading to larger *Q* factor for the lower frequency resonances (see Fig. 3b). We also observed an overall trend of increasing of *Q* factors with larger device sizes, consistent with the scaling rule of Eq. (2). Since in the presence of material damping all standing-wave modes will have similar material-absorption-limited *Q* factors in sufficiently large PnCs, we conclude that the observed resonances at room temperature are higher-order modes with significant radiation and scattering losses, while the lower-order modes with the more confined modal profile are obscured by the dominant substrate transmission background because of the much weaker external coupling comparing to their intrinsic losses.

### Radiation loss of mechanical BICs

**Fig. 4:**
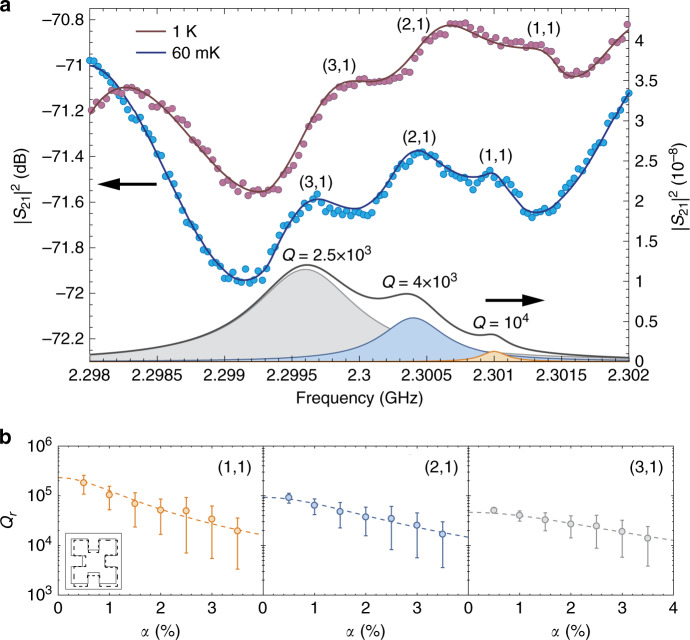
Radiative quality factor of mechanical BICs at cryogenic temperatures. **a** Transmission spectrum of a PnC with 200 × 200 unit cells measured at 1 K and 60 mK, showing lower-order *B*_1_–BIC standing-wave resonances. The right axis shows the transmission via the three resonances, superposed (black curve) and individual, at 60 mK by removing the substrate background. **b** Simulated radiative quality factor of the three BIC modes using unit cells with disorders in dimensions $b^{\prime}$s and $c^{\prime}$s. *α* is the standard deviation of relative variation. Dashed lines are global fitting using the model of Eq. (2). Error bar represents the standard deviation of *Q*_r_ (Supplementary Note 2).

To reveal the actual radiation loss of the lower-order BIC standing-wave resonances, we measured the sample at cryogenic temperatures, where material-absorption losses are suppressed. For example, in a PnC with 200 × 200 unit cells, we observed several lower-order *B*_1_-BIC standing-wave resonances emerging out of the substrate background (Fig. 4a). At *T* = 1 K, the three resonances, from high to low frequency, have *Q* factors of 5.2 × 10^3^, 2.8 × 10^3^, and 2.2 × 10^3^, respectively. At *T* = 60 mK, the *Q* factors further increase to 1.01 × 10^4^, 4.0 × 10^3^, and 2.5 × 10^3^, respectively. We found that the mechanical dissipation at 60 mK is dominated by radiation and scattering losses, while the two-level system driven damping^29,30^, which is the main anelastic loss at this temperature, is at least three orders weaker (Supplementary Note 2). As a result, the ratio between the *Q* factor of these resonances at 60 mK matches well to the scaling rule of Eq. (2), from which we determined the mode order of the three resonances to be (1, 1), (2, 1), and (3, 1), respectively (Supplementary Note 2). We simulated the radiative quality factor of these band-edge modes in the presence of structural disorders as shown in Fig. 4b, which is comparable to the measured *Q* factors after taking into account of the estimated scattering loss. Based on these results and the scaling rule of Eq. (2), we expect mechanical BICs with *Q* ≥ 10^7^ can be realized in millimeter-scale PnCs with improved fabrication and material quality, such as using single crystalline silicon^30^ or epitaxially grown materials^31–33^. The mechanical quality factor can also be further improved using the technique of topological charge merging to suppress scattering losses^19^.

## Discussion

In summary, we have shown a paradigm of phonon trapping via mechanical BICs in slab-on-substrate PnCs, revealing a fresh ground for studying BIC physics with unique features. The mechanical BIC is expected to enable a new breed of quantum mechanical oscillators with substrate-mediated heat capacity and dissipation of measurement-induced parasitic phonons. We envision several exciting research directions to be enabled by the mechanical BIC architecture. For example, microwave-frequency mechanical BICs in PnCs have an effective mass proportional to *N*^2^ that will approach milligrams in centimeter-scale PnCs (note *m*_eff_ = 0.51 pg for the 1 μm-size unit-cell *B*_1_–BIC mode), when cryogenically cooled to the quantum ground state (${\bar{n}}_{{\rm{th}}}\,\approx\,0.04$ phonons at 5 mK), representing an unparalleled platform for the exploration of macroscopic quantum mechanical effects, including testing wavefunction collapse via continuous spontaneous localization^34,35^. The slab-on-substrate architecture also allows integration with heterogeneous structures without introducing extra mechanical dissipations, which might enable unprecedented sensing modalities using high-*Q* mechanical BICs for direct detection of phonon generation caused by rare physical events in bulk acoustic matrices^36–38^.

## Methods

### Fabrication

Polycrystalline *c*-axis oriented AlN thin film is deposited on oxide silicon wafers via a sputtering process with dual cathode S-gun magnetron source. The AlN PnC is fabricated using electron beam lithography with chemical vapor deposited SiO_2_ and ZEP520A as masks, followed by inductively coupled plasma reactive ion etch (ICP-RIE) of oxide using CHF_3_ and another ICP-RIE of AlN using BCl_3_/Cl_2_/Ar. The chip is dipped in buffered hydrofluoric acid to remove the residual oxide mask of a few nanometers. The IDTs and electrodes are then fabricated using electron beam lithography and lift-off process with 100 nm evaporated aluminum.

### Measurement

Room-temperature transmission spectrum was measured using RF probes in contact with the on-chip electrodes. A vector network analyzer (VNA) was used for generating RF signals and receiving transmission spectrum. For cryogenic temperature device measurements, a printed circuit board is employed as mount and the chip was wire-bonded to it for electrical connection. The mount was placed in the mixing chamber of the dilution refrigerator. Strong attenuation was applied to the RF cables with 5 dBm output power from the VNA. The measurement was conducted in vacuum and low temperature (down to 60 mK).

### Transmission spectrum

The IDT-excited acoustic wave splits after encountering the PnC and the two parts propagate via the BIC mode in the slab and the SiO_2_ substrate mode, respectively. The two parts of the acoustic wave experience different losses and local environment and, as a result, develop a relative phase that fluctuates over time. The transmission coefficient *S*_21_ can be decomposed as *S*_21_ = *S*_*s*,21_ + *e*^iδφ^ *S*_*b*,21_, where *S*_*s(b)*,21_ is the transmission coefficient via the substrate(slab) and *δφ* is the relative phase between the two components. The VNA measures the averaged amplitude and phase of *S*_21_:

3 $$| {S}_{21}{| }^{2} 	=| {S}_{{{s}},21}{| }^{2}+| {S}_{{{b}},21}{| }^{2}+2{\langle \cos (\theta +\delta \varphi )\rangle }_{\delta \varphi }| {S}_{{{s}},21}| | {S}_{{{b}},21}| \\ 	\approx | {S}_{{{s}},21}{| }^{2}+| {S}_{{{b}},21}{| }^{2},$$

4 $${\rm{Arg}}[{S}_{21}]\approx {\rm{Arg}}[{S}_{{{s}},21}]+{\left\langle {\sin }^{-1}\left(\frac{| {S}_{{{b}},21}| }{| {S}_{{{s}},21}| }\sin (\theta +\delta \varphi )\right)\right\rangle }_{\delta \varphi },$$

where $\theta ={\rm{Arg}}[{S}_{{{b}},21}]-{\rm{Arg}}[{S}_{{{s}},21}]$ and 〈⋅〉_δφ_ means averaging over phase *δφ*. We have assumed $| {\langle \cos (\theta +\delta \varphi )\rangle }_{\delta \varphi }| \ll 1$ for quasi-random *δφ* in Eq. (3) and ∣*S*_*b*,21_∣ ≪ ∣*S*_*s*,21_∣ in Eq. (4) based on the simulated acoustic energy distribution of the AlN–SiO_2_ mode. Therefore, the phase of *S*_21_ is dominated by the substrate transmission, as observed in both room temperature and low temperature measurements.

*S*_*b*,21_ for a single BIC resonance can be derived using input–output formalism,

5 $$\frac{{\mathrm{d}}b}{{\mathrm{d}}t}=\left(-i{\omega }_{m}-\frac{\gamma }{2}\right)b+\sqrt{\frac{{\gamma }_{e}}{4}}{b}_{{\rm{in}}},\quad {b}_{{\rm{out}}}=\sqrt{\frac{{\gamma }_{e}}{4}}b,$$

which leads to

6 $${S}_{b,21}[\omega ]\equiv \frac{{b}_{{\rm{out}}}[\omega ]}{{b}_{{\rm{in}}}[\omega ]}=\frac{\frac{{\gamma }_{e}}{4}}{i({\omega }_{m}-\omega )+\frac{\gamma }{2}},$$

where *b* is the mode amplitude of the BIC, *γ* ≡ *ω*_*m*_/*Q* and *γ*_*e*_ ≡ *ω*_*m*_/*Q*_*e*_. Fitting of the measured transmission spectrum thus uses Eq. (3) with ∣*S*_*s*,21_∣^2^ a cubic function and ∣*S*_*b*,21_∣^2^ comprising multiple Lorentzian functions corresponding to all resolvable peaks, i.e.,

7 $$| {S}_{21,{\rm{fit}}}[\omega ]{| }^{2}=\mathop{\sum }\limits_{n=0}^{3}{a}_{{{n}}}{\omega }^{n}+\eta \sum _{{{j}}}\frac{{\left(\frac{{\gamma }_{{{e,j}}}}{4}\right)}^{2}}{{(\omega -{\omega }_{{{m,j}}})}^{2}+{\left(\frac{{\gamma }_{{{j}}}}{2}\right)}^{2}},$$

where variables besides *ω* are all fitting parameters and *η* accounts all other losses in the circuit. Quantitatively, the relative standard deviation of the fitting is typically less than 1%. From Eq. (7), we are able to infer *γ*_e_ relative to one reference resonance as shown in Fig. 3d, e. Regarding the phase of *S*_21_, Eq. (4) applies to the general case of multiple resonances, where *S*_b,21_ consists of multiple functions of Eq. (6).

## Supplementary information

Supplementary Information

## Data Availability

Data supporting the findings of this study are available within the article and its Supplementary Information, or from the corresponding author upon reasonable request.
