# Supplementary material for: Observation of phonon trapping in the continuum with topological charges
Source: Nat Commun. 2020 Oct 15;11:5216. doi: 10.1038/s41467-020-19091-3 (PMC7567064; doi:10.1038/s41467-020-19091-3)
Supplement: Supplementary file 1 — Supplementary Information [file 41467_2020_19091_MOESM1_ESM.pdf]

Supplementary Information for "Observation of phonon trapping in the continuum with topological charges"

Hao Tong,[1, 2, *] Shengyan Liu,[1, 2, *] Mengdi Zhao,[1, 2] and Kejie Fang[1, 2, †]

[1] *Holonyak Micro and Nanotechnology Laboratory and Department of Electrical and Computer Engineering, University of Illinois at Urbana-Champaign, Urbana, IL 61801 USA*

[2] *Illinois Quantum Information Science and Technology Center, University of Illinois at Urbana-Champaign, Urbana, IL 61801 USA*

SUPPLEMENTARY NOTE 1: TRANSVERSE TOPOLOGICAL CHARGES AND ACCIDENTAL MECHANICAL BICS

A. Definition of transverse topological charges

We define a topological charge associated with mechanical BICs, based on the far-field transverse polarization. We decompose the far-field amplitude as $\mathbf{c} = \mathbf{c}_T + \mathbf{c}_L = c_T\mathbf{e}_T + c_L\mathbf{e}_L$, where $\mathbf{e}_{T,L}$ are unit vectors along the transverse and longitudinal polarization directions (Supplementary Figure 2a). It is straightforward to show

$$\mathbf{c}_L = \frac{\mathbf{c}\cdot\mathbf{k}_T}{\mathbf{e}_L\cdot\mathbf{k}_T}\mathbf{e}_L,\ \mathbf{c}_T = \mathbf{c} - \mathbf{c}_L, \tag{1}$$

where $\mathbf{k}_T \perp \mathbf{e}_T$ is the wavevector of the transverse far-field.

From the transverse radiation field $\mathbf{c}_T(\mathbf{k})$, we define the topological charge of a Bloch mode to be

$$q = \frac{1}{2\pi}\oint_C d\mathbf{k}\cdot\nabla_{\mathbf{k}}\theta(\mathbf{k}), \tag{2}$$

where C is a path enclosing the Bloch mode in Brillouin zone and θ is the angle between the major axis of the elliptical polarization and the k_x axis. Here, we define the major axis $\mathbf{A_c}$ to be that of the in-plane polarization vector $\mathbf{c}_{T,\parallel}$ [1], i.e., the projection of $\mathbf{c}_T$ onto the x-y plane,

$$\mathbf{A_c} = \frac{1}{|\sqrt{\mathbf{c}_{T,\parallel}\cdot\mathbf{c}_{T,\parallel}}|}\,\mathrm{Re}\left[\mathbf{c}_{T,\parallel}\sqrt{\mathbf{c}^*_{T,\parallel}\cdot\mathbf{c}^*_{T,\parallel}}\right]. \tag{3}$$

Based on this definition, we calculated the in-plane polarization field distribution and topological charge of the three mechanical BICs at the Γ point, as shown in Supplementary Figure 1d. All of them are associated with integer charges. The topological charge of mechanical BICs might be measured using laser Doppler vibrometer to map out the vibration at the SiO_2-Si interface when the mechanical BIC is excited.

B. Accidental BICs on high-symmetric lines

Besides the BICs at high-symmetric points, there could be accidental BICs at regular $\boldsymbol{k}$ points in the first Brillouin zone. One argument for this is based on radiation amplitude cancellation. In the optical case, since electromagnetic fields only have transverse polarization, it is possible to tune the real far-field radiation amplitude $(c_x(k_x,k_y), c_y(k_x,k_y))$ to be zero for some Bloch wavevector (k_x,k_y) in z-symmetric structures [2]. However, for the acoustic case, because of the existence of both transverse and longitudinal waves and they have different sound speed, in general it is impossible to tune $(c_x(k_x,k_y), c_y(k_x,k_y), c_z(k_x,k_y))$ to be all zero with only two variables k_x and k_y.

It turns out, with some additional symmetry constraints, it is possible to realize accidental mechanical BICs. For this purpose, we consider potential accidental BICs on the high-symmetric lines, i.e., $\Gamma\to X$ and $\Gamma\to M$. For Bloch modes on the high-symmetric lines, they belong to the representation A' and A'' of C_{1h} group, which are even and odd

* These authors contributed equally to this work.
† kfang3@illinois.edu

under the mirror operation about the line, respectively. For the far-field longitudinal polarization to vanish, the mode has to be odd. This also dictates the far-field transverse polarization to be perpendicular to the mirror plane. As a result, for modes on the high-symmetric lines, their far-field radiation amplitude only has one transverse component, which can be chosen to be real following the argument of Supplementary Equations 9 and 10. Since phonons only propagate in solids, thus there is only downward acoustic radiation in the slab-on-substrate structures. Thus, by varying the Bloch wavevector along the high-symmetric line, it is possible to eliminate this only component to realize accidental mechanical BICs.

Supplementary Table 1. Representation reduction of C_{4v} group along $\Gamma \to X$ and $\Gamma \to M$

C_{4v}	$C_{1h}(\Gamma \to X)$	$C_{1h}(\Gamma \to M)$
A_1	A'	A'
A_2	A''	A''
B_1	A'	A''
B_2	A''	A'
E	$A' + A''$	$A' + A''$

Based on this argument, we examine possible accidental mechanical BICs in structures with C_{4v} symmetry and their relation to the modes at the Γ point. As the wavevector moves away from the Γ point along the high-symmetric lines $\Gamma \to X$ and $\Gamma \to M$, the representations of C_{4v} group at the Γ point reduces to representations of C_{1h} group, as summarized in Supplementary Table 1. Only modes with the A'' representation, i.e., odd under mirror operation, could possibly be a mechanical BIC. In Supplementary Figure 1, we illustrate, specifically for A_2, B_2, and B_1 modes at the Γ point which are symmetry-induced BICs, high-symmetric lines along which they could evolve to accidental BICs.

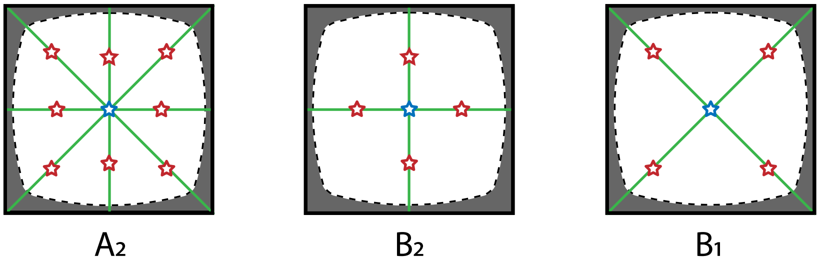

Supplementary Figure 1. Possible accidental mechanical BICs (red star) associated with symmetry-induced BICs (blue star) at the Γ point. Green lines indicate the nodal lines of longitudinal radiation amplitude.

C. Numerical demonstration of accidental BICs

For the structure studied in the main text, we swept the mechanical bands along the high-symmetric lines, $\Gamma \to X$ and $\Gamma \to M$, and find high-Q mechanical modes. We also calculated the transverse far-field polarization which reveals the topological properties of these high-Q modes. It turns out that the high-Q mode on the $\Gamma \to X$ line is associated with two half-charges, i.e., $q = 1/2$, aside of the x-axis (Supplementary Figure 2b), while the one on the $\Gamma \to M$ line is an accidental BIC with an integer charge (Supplementary Figure 2c) which is evolved from a mode belong to the E representation at the Γ point.

We also characterized the degree of circular polarization near the high-Q modes. The far-field transverse polarization of the Bloch mode can be decomposed in the circular polarization basis, i.e.,

$$\mathbf{c}_{T,||} = \alpha_R \mathbf{c}_R + \alpha_L \mathbf{c}_L, \tag{4}$$

and the degree of circular polarization is defined as

$$m_c = \frac{|\alpha_R|^2 - |\alpha_L|^2}{|\alpha_R|^2 + |\alpha_L|^2} \in [-1, 1]. \tag{5}$$

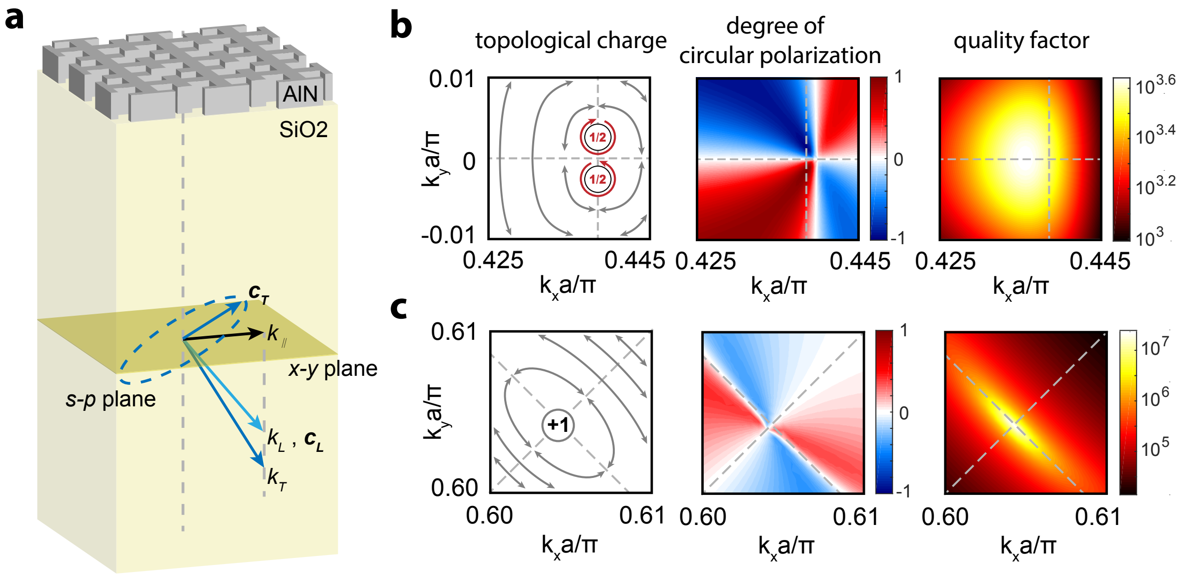

Supplementary Figure 2. **a**, Schematics of decomposition of far-field radiation amplitude. **b**, Half-charge high-Q modes along the $\Gamma \to X$ line. Left panel: transverse polarization vector field and topological charge. Middle panel: degree of circular polarization. Right panel: quality factor. **c**, Accidental BIC with integer topological charge on the $\Gamma \to M$ line.

As seen from Supplementary Figure 2c, the accidental BIC is located at the intersection of two perpendicular lines with $m_c = 0$ corresponding to linear polarizations. The quality factor of the accidental mechanical BIC with non-zero wavevectors is less than the BICs at the Γ point. We find this might just be numerical manifestation due to the simulation software COMSOL, whose default acoustic PML/low-reflection boundary conditions do not work perfectly for modes with large transverse momentum, because it turns out that mechanical modes below the acoustic line are also missing ultrahigh Q factors.

We find the accidental BIC on the $\Gamma \to M$ line persists as we vary the thickness of the AlN slab. The robust existence of the accidental BIC in certain structural parameter range can be argued as follows. For a given unit cell structure, we vary one specific parameter, e.g., the thickness of slab h. The transverse polarization field c_T perpendicular to the high-symmetric line, which is chosen to be real, is a function of h and k along the high-symmetric line. *If* there exists an accidental BIC for some h_0 and k_0, i.e., $c_T(h_0, k_0) = 0$, and since the zeros of the 2D function $c_T(h, k)$ in general consists of a 1D curve in the h-k plane, one can always find k' satisfying $c_T(h', k') = 0$ for arbitrary h' around h_0.

SUPPLEMENTARY NOTE 2: QUALITY FACTOR OF MECHANICAL BICS

A. Radiative quality factor: symmetry-preserving structure

We derive the radiative quality factor of Bloch modes in the vicinity of mechanical BICs at the Γ point. The mechanical Bloch modes satisfy the following eigenequation

$$-\omega^2 \rho(\mathbf{r}) Q_i(\mathbf{r}) = \partial_j \left(C_{ijkl}(\mathbf{r}) \partial_k Q_l(\mathbf{r}) \right), \tag{6}$$

where $\rho(\mathbf{r})$ is the density, $C_{ijkl}(\mathbf{r})$ is the elasticity tensor, and $Q_l(\mathbf{r}) = e^{i\mathbf{k}\cdot\mathbf{r}} u_{\mathbf{k}l}(\mathbf{r})$ is the displacement field of the Bloch mode. For a transversely isotropic material system, such as the AlN-on-oxide system studied in this paper, the elastic tensor takes the form

$$C = \begin{bmatrix} C_{1111} & C_{1122} & C_{1133} & 0 & 0 & 0 \\ & C_{1111} & C_{1133} & 0 & 0 & 0 \\ & & C_{3333} & 0 & 0 & 0 \\ & & & C_{2323} & 0 & 0 \\ & & symm & & C_{2323} & 0 \\ & & & & & \frac{1}{2}\left(C_{1111} - C_{1122}\right) \end{bmatrix}. \tag{7}$$

When the system possesses $C_2^z T$ symmetry, i.e., $C^*_{ijkl}(x,y,z) = C_{ijkl}(-x,-y,z)$ and $\rho^*(x,y,z) = \rho(-x,-y,z)$, we find that for an eigenfunction $\mathbf{u_k}(\mathbf{r})$ of Supplementary Equation 6,

$$C_2^z \mathbf{u}^*_\mathbf{k}\left(C_2^z \mathbf{r}\right) = \big(-u^*_{\mathbf{k},1}(-x,-y,z), -u^*_{\mathbf{k},2}(-x,-y,z), u^*_{\mathbf{k},3}(-x,-y,z)\big) \tag{8}$$

is also an eigenfunction with the same eigenvalue. Thus, $\mathbf{u_k}(\mathbf{r})$ and $C_2^z \mathbf{u}^*_\mathbf{k}\left(C_2^z \mathbf{r}\right)$ must differ at most by an arbitrary phase factor, which can be chosen to be -1 for all $\mathbf{k}$, i.e.,

$$\big(u_{\mathbf{k},1}(x,y,z), u_{\mathbf{k},2}(x,y,z), u_{\mathbf{k},3}(x,y,z)\big) = \big(u^*_{\mathbf{k},1}(-x,-y,z), u^*_{\mathbf{k},2}(-x,-y,z), -u^*_{\mathbf{k},3}(-x,-y,z)\big). \tag{9}$$

The far-field radiation amplitude of the Bloch mode is characterized by the averaged value of $\mathbf{u_k}(\mathbf{r})$ in the $x-y$ plane, i.e.,

$$\left(c_x(\mathbf{k}), c_y(\mathbf{k}), c_z(\mathbf{k})\right) \equiv \left(\langle u_{\mathbf{k},1}\rangle_{xy}, \langle u_{\mathbf{k},2}\rangle_{xy}, \langle u_{\mathbf{k},3}\rangle_{xy}\right), \tag{10}$$

Thus, according to Supplementary Equation 9, $c_x(\mathbf{k})$ and $c_y(\mathbf{k})$ are real while $c_z(\mathbf{k})$ is pure-imaginary.

Next we relate the eigenfunction at $\mathbf{k}$ and the eigenfunction at $\mathcal{R}\mathbf{k}$, where $\mathcal{R}$ is an element of the point group of the structure. Since the structure is invariant under transformation $\mathcal{R}$, we know $\hat{O}_\mathcal{R}\mathbf{u_k}$ is also an eigenfunction of Supplementary Equation 6 with Bloch wavevector $\mathcal{R}\mathbf{k}$. In the case without degeneracy, we have $\hat{O}_\mathcal{R}\mathbf{u_k} = \alpha_\mathbf{k}\mathbf{u}_{\mathcal{R}\mathbf{k}}$. Since $\mathbf{u_k}$ is chosen to fulfill the realness of $c_{x,y}$ and ic_z, and $\hat{O}_\mathcal{R}$ does not mix x,y-components with z-component for a 2-D point group, $\alpha_\mathbf{k}$ has to be real. When $\mathbf{u_k}$ is normalized, we obtain $\alpha_\mathbf{k} = \pm 1$. Because $\alpha_\mathbf{k}$ is also continuous regarding $\mathbf{k}$, it has to be a constant (1 or -1), which thus equals α_Γ. The latter is related to the spatial symmetry of the mode at the Γ point, i.e., $\hat{O}_\mathcal{R}\mathbf{u}_\Gamma = \alpha_\Gamma\mathbf{u}_\Gamma$ (since $\mathcal{R}\Gamma = \Gamma$). In conclusion, we have

$$\mathbf{u}_{\mathcal{R}\mathbf{k}} = \alpha_\Gamma \hat{O}_\mathcal{R}\mathbf{u_k}. \tag{11}$$

And the averaged far-field amplitude of Supplementary Equation 10 obeys the same relation. This result is identical to the optical case [3].

Using Supplementary Equation 11, we can find the asymptotic expression of the far-field amplitude of the Bloch modes in the vicinity of BICs at the Γ point. The averaged far-field amplitude of the Bloch modes in the vicinity of BICs can be expanded around $\mathbf{k} = 0$ as:

$$c_x(\mathbf{k}) = \sum_{\substack{m,n\geq 0\\ m+n\geq 1}} p_{mn}k_x^m k_y^n,\ c_y(\mathbf{k}) = \sum_{\substack{m,n\geq 0\\ m+n\geq 1}} q_{mn}k_x^m k_y^n,\ c_z(\mathbf{k}) = \sum_{\substack{m,n\geq 0\\ m+n\geq 1}} r_{mn}k_x^m k_y^n, \tag{12}$$

because at $\mathbf{k} = 0$, $c_x(0) = c_y(0) = c_z(0) = 0$ for BICs. Taking the B_1 BIC mode as an example, it is even under mirror operation $\sigma_{x,y}$, i.e., $\mathbf{u}_{\sigma_{x,y}\mathbf{k}} = \sigma_{x,y}\mathbf{u_k}$, so we have

$$\begin{aligned} c_x\left(k_x,k_y\right) &= c_x\left(k_x,-k_y\right) = -c_x\left(-k_x,k_y\right),\\ c_y\left(k_x,k_y\right) &= -c_y\left(k_x,-k_y\right) = c_y\left(-k_x,k_y\right),\\ c_z\left(k_x,k_y\right) &= c_z\left(k_x,-k_y\right) = c_z\left(-k_x,k_y\right). \end{aligned} \tag{13}$$

As a result, only those terms in Supplementary Equation 12 respecting the symmetry constraint of Supplementary Equation 13 will be present, i.e., to the leading order

$$\begin{aligned} c_x\left(k_x,k_y\right) &= p_1k_x + p_2k_x^3 + p_3k_xk_y^2 + O\left(k^5\right),\\ c_y\left(k_x,k_y\right) &= q_1k_y + q_2k_y^3 + q_3k_yk_x^2 + O\left(k^5\right),\\ c_z\left(k_x,k_y\right) &= r_1k_x^2 + r_2k_y^2 + r_3k_x^4 + r_4k_y^4 + r_5k_x^2k_y^2 + O\left(k^6\right). \end{aligned} \tag{14}$$

The B_1 BIC is also odd under $\frac{\pi}{2}$ rotation around the z-axis, i.e., $\mathbf{u}_{\mathcal{R}_{\frac{\pi}{2}}\mathbf{k}} = -\mathcal{R}_{\frac{\pi}{2}}\mathbf{u_k}$, which leads to

$$\begin{aligned} c_x\left(-k_y,k_x\right) &= c_y\left(k_x,k_y\right),\\ c_y\left(-k_y,k_x\right) &= -c_x\left(k_x,k_y\right),\\ c_z\left(-k_y,k_x\right) &= -c_z\left(k_x,k_y\right). \end{aligned} \tag{15}$$

Supplementary Equation 15 further constrains the coefficients in Supplementary Equation 14, leading to $p_{1,2,3} =$

$-q_{1,2,3}$, $r_{1,3}=-r_{2,4}$, and $r_5=0$. In conclusion, the radiation field of the Bloch modes near the B_1 mechanical BIC is given by

$$\begin{aligned} c_x\left(k_x,k_y\right) &= p_1 k_x + p_2 k_x^3 + p_3 k_x k_y^2 + O\left(k^5\right), \\ c_y\left(k_x,k_y\right) &= -p_1 k_y - p_2 k_y^3 - p_3 k_y k_x^2 + O\left(k^5\right), \\ c_z\left(k_x,k_y\right) &= r_1(k_x^2-k_y^2) + r_3(k_x^4-k_y^4) + O\left(k^6\right). \end{aligned} \tag{16}$$

Similarly, we find for Bloch modes in the vicinity of the B_2 BIC,

$$\begin{aligned} c_x\left(k_x,k_y\right) &= p_1 k_y + p_2 k_y^3 + p_3 k_x^2 k_y + O\left(k^5\right), \\ c_y\left(k_x,k_y\right) &= p_1 k_x + p_2 k_x^3 + p_3 k_x k_y^2 + O\left(k^5\right), \\ c_z\left(k_x,k_y\right) &= r_1 k_x k_y + r_2 k_x k_y\left(k_x^2+k_y^2\right) + O\left(k^6\right), \end{aligned} \tag{17}$$

and of the A_2 BIC,

$$\begin{aligned} c_x\left(k_x,k_y\right) &= p_1 k_y + p_2 k_y^3 + p_3 k_x^2 k_y + O\left(k^5\right), \\ c_y\left(k_x,k_y\right) &= -p_1 k_x - p_2 k_x^3 - p_3 k_x k_y^2 + O\left(k^5\right), \\ c_z\left(k_x,k_y\right) &= r_2 k_x k_y\left(k_y^2-k_x^2\right) + O\left(k^6\right). \end{aligned} \tag{18}$$

The radiative quality factor of the Bloch mode is inversely proportional to the outflow acoustic energy flux. The acoustic energy flux $\mathbf{P}$ is defined as [4]

$$\mathbf{P} = -\boldsymbol{\tau}\dot{\mathbf{Q}}^*, \tag{19}$$

where the stress tensor τ_{ij} is given by Hooke's law: $\tau_{ij}=c_{ijkl}\left(\partial_k Q_l + \partial_l Q_k\right)/2$. Thus, the z-component of the energy flux in a transversely isotropic medium is given by

$$P_z = -\lambda \dot{Q}_x^* \partial_z Q_x - \lambda \dot{Q}_y^* \partial_z Q_y - (\lambda+2\mu)\dot{Q}_z^* \partial_z Q_z, \tag{20}$$

where λ and μ is Lamé's first and second parameter, respectively. For Bloch modes near the Γ point,

$$P_z \approx \lambda \frac{\omega^2}{v_T}(|c_x|^2+|c_y|^2) + (\lambda+2\mu)\frac{\omega^2}{v_L}|c_z|^2, \tag{21}$$

where ω is the frequency of the Bloch mode and $v_{T(L)}$ is the sound speed of the transverse(longitudinal) plane-wave. Since $\omega=\omega_0+O(k)$, the dependence of radiative quality factor Q_r on $\mathbf{k}$, to the leading order, is found to be

$$Q_r \propto \frac{1}{P_z} \propto \frac{1}{|c_x|^2+|c_y|^2+\beta|c_z|^2}, \tag{22}$$

where $\beta=(\lambda+2\mu)v_T/\lambda v_L$, which in conjunction with Supplementary Equations 16, 17, or 18 yields

$$Q_r \propto \frac{1}{k_x^2+k_y^2}, \tag{23}$$

where we have assumed the leading terms in c_z is much smaller than the leading terms in $c_{x,y}$. This is confirmed with the numerical simulation shown in Supplementary Figure 3.

B. Radiative quality factor: symmetry-breaking perturbations

When the unit cell acquires symmetry-breaking perturbations, the mechanical BICs at the Γ point couple into the radiation continuum and become quasi-BICs with finite radiative quality factor. We assume the perturbation can be characterized by a perturbation parameter α. The far-field amplitude of the quasi-BIC now contains all the terms of Supplementary Equation 12 in addition to constants, with the coefficients of these terms being functions of α. However, to the leading order of both k and α, the far-field amplitude can be approximated by only a few terms. For

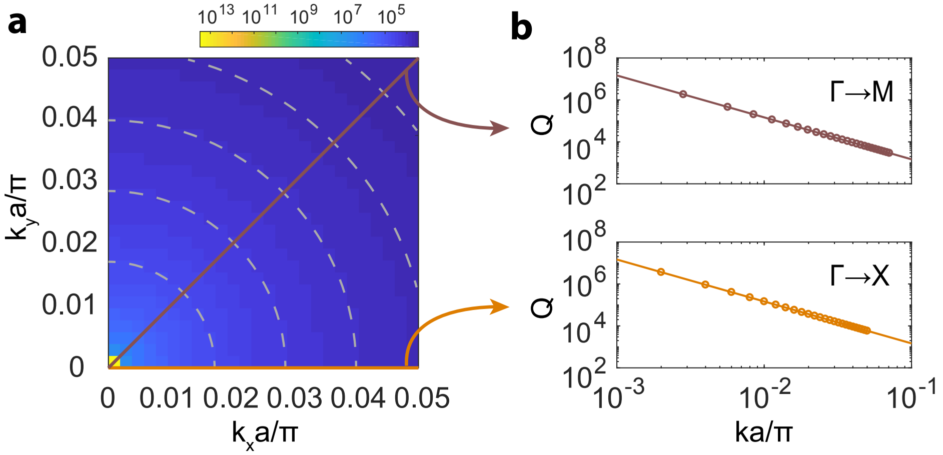

Supplementary Figure 3. **a**, Simulated radiative quality factor of Bloch modes in the vicinity of B_1 BIC at the Γ point. Dashed lines are equi-Q factor contours. **b**, Two cut lines along $\Gamma - M$ and $\Gamma - X$ direction. Solid lines show the scaling rule of $Q_r \propto 1/k^2$.

example, for the B_1 quasi-BIC, we have

$$\begin{aligned} c_x(k_x, k_y) &\approx p_0\alpha + p_1 k_x, \\ c_y(k_x, k_y) &\approx q_0\alpha - p_1 k_y, \\ c_z(k_x, k_y) &\approx i r_0 \alpha, \end{aligned} \tag{24}$$

which, according to Supplementary Equation 22, leads to

$$Q_r \propto \frac{1}{(p_0\alpha + p_1 k_x)^2 + (q_0\alpha - p_1 k_y)^2 + \beta r_0^2 \alpha^2}, \tag{25}$$

$$\propto \frac{1}{k_x^2 + k_y^2 + \zeta'\alpha^2 + 2\alpha p_1^{-1}(p_0 k_x - q_0 k_y)}, \tag{26}$$

where $\zeta' = (p_0^2 + q_0^2 + \beta r_0^2)/p_1^2 > 0$. Since we are considering the scaling rule of the statistical average of Q_r for a class of perturbations corresponding to the same parameter α, the first moment of the independent p's and q's is zero, and thus the last term in the denominator of Supplementary Equation 26 vanishes. Thus,

$$Q_r \propto \frac{1}{k_x^2 + k_y^2 + \zeta'\alpha^2} \tag{27}$$

$$\propto \left(\frac{n^2 + m^2}{N^2} + \zeta\alpha^2\right)^{-1}. \tag{28}$$

At the Γ point, $Q_r \propto \alpha^{-2}$, which is consistent with the result of Ref. [5].

C. Scattering loss

We provide a phenomenological analysis of the phonon scattering loss. The slight inhomogeneity of unit cells in actual PnCs causes weak scattering of phonons among isofrequency modes, which all have roughly the same radiative quality factor (Supplementary Equation 28). As a consequence, the radiation loss of a band-edge mode is increased by a factor roughly equal with the number of isofrequency modes it can scatter to, according to Fermi's golden rule, which resembles Purcell's effect [6]. For relatively weak disorders, the dominant scattering process involves isofrequency modes in the vicinity of the mode under consideration because of large field-overlap and the mode with opposite momentum because of time-reversal symmetry induced coherent backscattering [7], as shown in Supplementary Figure 4. As a result, the radiation enhancement factor is approximately an $O(1)$ constant λ for the

modes in the vicinity of the Γ point, i.e.,

$$\frac{1}{Q_r} + \frac{1}{Q_s} \approx \frac{\lambda}{Q_r}. \tag{29}$$

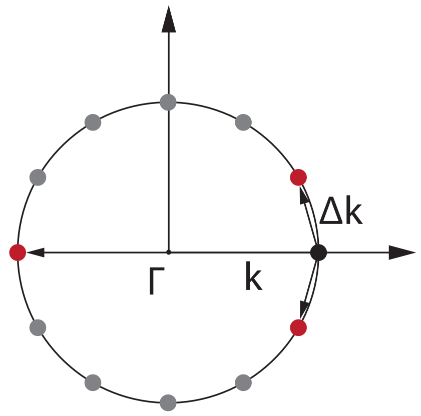

Supplementary Figure 4. Illustration of mode scattering on an isofrequency contour close to the Γ point, dominated by scattering to the modes labeled by red dots.

D. Material losses

Now we consider material-related losses and show they are all ignorable at the lowest temperature (60 mK) measured in this work, comparing to the acoustic radiation and scattering losses.

Phonon-phonon interaction

At low temperature when $\omega_m \tau \gg 1$, τ the thermal phonon relaxation time, the dominant phonon-phonon interaction is the Landau-Rumer effect, where acoustic phonons interact with individual thermal phonons. The effect is very weak though as experimentally showed, for example, in silicon optomechanical cavities [8, 9], leading to the characterizing Q factor $Q_{a,\text{ph-ph}} > 10^{13}$ below 1 K. We expect similar behavior of phonon-phonon interaction in AlN, with $Q_{a,\text{ph-ph}}$ at least a few orders larger than the measured Q factor of the BIC standing-wave modes at low temperature ($\sim 10^4$).

Grain-boundary relaxation

The AlN used in this work is polycrystalline with grain size about 50 nm based on scanning electron microscopy. These grains are still pretty well aligned with x-ray diffraction analysis showing a rocking curve angle $< 1.5°$. A natural speculation of another possible material loss is thus the grain-boundary relaxation. However, this effect is usually observed at elevated temperatures. Taking typical high purity metal as a reference, the grain-boundary relaxation activation energy $\Delta\epsilon \sim 0.1$ eV [10] which is four orders larger than $k_\text{B}T$ for $T = 60$ mK, leading to ultra-long relaxation time $\tau = \tau_0 e^{\frac{\Delta\epsilon}{k_\text{B}T}}$ ($\tau_0 \approx 10^{-12}$–10^{-10} s), irrelevant to the mechanical damping at such low temperature.

Two-level state

Another common source of mechanical dissipation is via coupling between phonons and two-level states (TLS)–a generic defect state in a solid-state material which possesses two local arrangements of atoms with nearly degenerate energy and is associated with both electric and acoustic dipoles. TLS has been found as the dominant source for acoustic damping below 1 K in both single crystalline materials [9, 11] and vitreous glass [12]. For the polycrystalline AlN here, where TLS tends to aggregate on the grain boundary, we expect TLS-induced loss is also the dominant material-related acoustic loss at low temperature.

For high frequency, microwave phonon modes and TLS at cryogenic temperatures, and assuming dispersive phonon-

TLS coupling, it is shown that the TLS-induced dissipation of a mechanical mode is given by [9, 12].

$$\gamma_{a,\mathrm{TLS}} = \sum_{\mathrm{TLS}} f\left[\mathbf{r}_{\mathrm{TLS}}\right] \frac{\omega_{\mathrm{TLS}}^d}{T} \operatorname{csch}\left[\hbar\omega_{\mathrm{TLS}}/k_B T\right], \tag{30}$$

where d is the dimension of the phonon bath, ω_{TLS} is the frequency splitting of the TLS, and $f\left[\mathbf{r}_{\mathrm{TLS}}\right]$ is a function of the spatial location of TLS and parameters other than T and ω_{TLS}, including TLS-phonon coupling rate, material density, and sound speed. For a uniform spectral and spatial density of states for the TLS, performing the integration in Supplementary Equation 30 yields

$$\gamma_{a,\mathrm{TLS}} \propto n_{0,\mathrm{TLS}} V_m T^d \tag{31}$$

where $n_{0,\mathrm{TLS}}$ is the number of TLS per frequency per volume and V_m is the acoustic mode volume.

First, we verify that the measured material-related quality factor of the BICs at 1 K is consistent with the TLS-induced loss in the AlN PnC by comparing with the result of Ref. [9]. Ref. [9] measured the Q factor of a 5 GHz breathing mode of a silicon optomechanical crystal nanobeam to be $Q_{\mathrm{Si}} \approx 2 \times 10^7$ at 1 K, and found it is limited by TLS-induced loss with $n_{0,\mathrm{TLS}}$=692.8 states/μm^3/GHz (taken from the value for the vitreous glass [12]) and etching-damaged, TLS-populated volume $V_{\mathrm{Si},m} = 0.039$ μm^3. If we assume the TLS density for the polycrystalline AlN to be one order smaller than the vitreous glass (note single crystalline silicon is estimated to have TLS density two orders smaller than the vitreous glass [11]), and consider the B_1 BIC with unit-cell mode volume $V_{\mathrm{BIC},m}$=0.156 μm^3, then in a PnC with 200×200 unit cells, the TLS-loss limited quality factor is roughly $Q_a \approx \frac{V_{\mathrm{Si},m}}{200^2 \times V_{\mathrm{BIC},m}} Q_{\mathrm{Si}} \approx 10^3$, according to Supplementary Equation 31. This is actually close to the measured Q_a for the BIC standing-wave modes at 1 K, which is $1/(1/Q_{1\mathrm{K}} - 1/Q_{60\mathrm{mK}}) \approx 10^4$, assuming $Q_{60\mathrm{mK}}$ is merely due to radiation and scattering loss which is to be verified right below. Now, taking the measured TLS-loss limited $Q_{a,\mathrm{TLS}} \approx 10^4$ at 1 K, using the scaling rule of Supplementary Equation 31, and given $d = 3$ for our slab-on-substrate structure, we have

$$Q_{a,\mathrm{TLS}} \approx 10^4 \times \left(\frac{1\ \mathrm{K}}{60\ \mathrm{mK}}\right)^3 \approx 4.6 \times 10^7 \tag{32}$$

at 60 mK, which is three orders larger than the measured Q factor.

E. Quality factor at base temperature

Based on the analysis above, the quality factor of the mechanical BIC modes at 60 mK is dominated by the radiation and scattering losses, i.e.,

$$\frac{1}{Q} \approx \frac{1}{Q_r} + \frac{1}{Q_s} + \frac{1}{Q_e}. \tag{33}$$

We also numerically confirmed that $Q_e > 10^5$ for the fundamental mode in y-periodic PnCs with $N = 50$ along the x-axis, and Q_e increases with N, so it can be ignored comparing to the Q factor measured at 1 K and 60 mK. Using these, we are able to determine the mode order of the three resonances of the PnC with 200×200 unit cells observed at the base temperature. First, we assume the mode order along the y-direction to be $m = 1$. This is because the $m = 2$ modes are odd with respect to the $x-$axis and cannot couple with the incident wave, and the coupling of higher order even modes with the incident wave roughly scales as $1/m$ due to field oscillation along the y-axis, which leads to power transmission that scales as $1/m^2$, leaving these resonances too weak to be detected. Thus, we assume the mode order of the three resonances to be $(n, 1), (n + 1, 1), (n + 2, 1)$. According to Supplementary Equations 28 and 29, the ratio between the quality factor of these resonances is

$$Q_n : Q_{n+1} : Q_{n+2} = \frac{1}{\frac{n^2+1}{N^2} + \zeta\alpha^2} : \frac{1}{\frac{(n+1)^2+1}{N^2} + \zeta\alpha^2} : \frac{1}{\frac{(n+2)^2+1}{N^2} + \zeta\alpha^2}. \tag{34}$$

Thus,

$$\frac{Q_n}{Q_{n+1}} < \frac{(n+1)^2+1}{n^2+1} \leq 2, \quad n \geq 2 \tag{35}$$

and

$$\frac{Q_n}{Q_{n+2}} < \frac{(n+2)^2+1}{n^2+1} \leq 3.4, \quad n \geq 2 \tag{36}$$

The measured values, however, are $\frac{Q_n}{Q_{n+1}} = 2.5$ and $\frac{Q_n}{Q_{n+2}} = 4.0$, which leads to the only option $n = 1$. These lower order modes indeed have much weaker external coupling comparing to the measured radiation loss, consistent with the deduction above where $1/Q_e$ has been ignored.

F. Simulation of radiation loss

To simulate the radiation loss of BIC standing-wave resonances in structures with imperfection, we introduced variations of the dimension b's and c's in the unit cell. Each b and c in the unit cell is varied independently, constrained by percentage standard deviation α. For each α, we simulated 50 unit-cell structures with randomly generated b's and c's, and by selecting the Bloch wavevector corresponding to the standing-wave mode, calculated the radiative quality factor of the latter. The average of Q_r, and the average and standard deviation of loss rate $\gamma_r \equiv \omega_m/Q_r$ are directly calculated. The standard deviation of Q_r is calculated using $\sigma(Q_r) \equiv \sigma(\gamma_r)/\bar{\gamma}_r \cdot \bar{Q}_r$. $\bar{Q}_r$ and $\sigma(Q_r)$ are plotted in Supplementary Figure 4b.

The simulated radiative quality factor of $(1,1)$, $(2,1)$, and $(3,1)$ modes in the 200×200 PnC versus disorders, as shown in Supplementary Figure 4b, is then fitted globally using the formula

$$Q_r = A\left(\frac{n^2+m^2}{N^2} + \zeta\alpha^2\right)^{-1}, \tag{37}$$

where both A and ζ are global fitting parameters, i.e., they are the same for the three modes.

SUPPLEMENTARY NOTE 3: BROADBAND TRANSMISSION SPECTRUM OF PHONONIC CRYSTALS

Supplementary Figure 5 shows a broadband transmission spectrum of the phononic crystal corresponding to Supplementary Figure 3a, which verifies the absence of B_2 modes because of the symmetry incompatibility with the excitation waves.

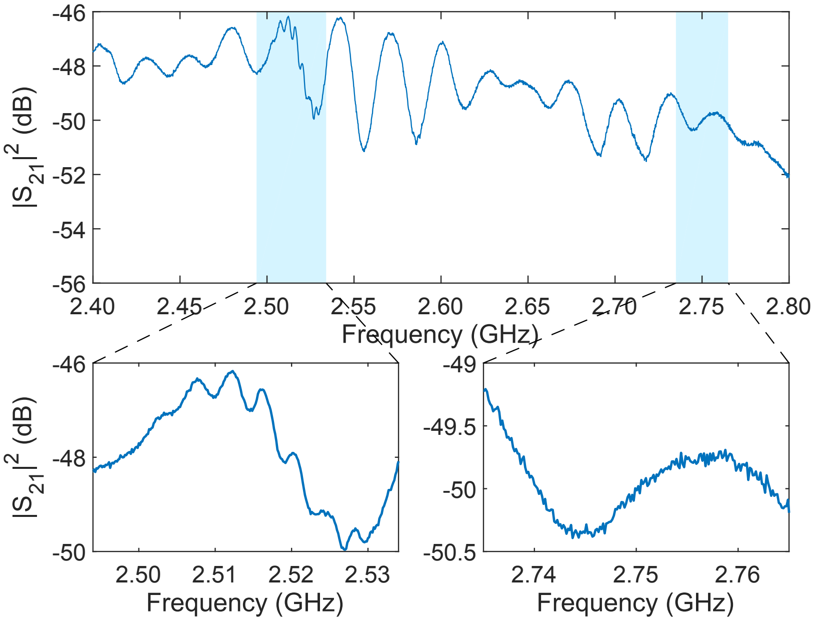

Supplementary Figure 5. Broadband transmission spectrum.

SUPPLEMENTARY REFERENCES

[1] Berry, M. Index formulae for singular lines of polarization. *Journal of Optics A: Pure and Applied Optics* **6**, 675 (2004).

[2] Hsu, C. W., Zhen, B., Lee, J., Chua, S.-L., Johnson, S. G., Joannopoulos, J. D. & Soljačić, M. Observation of trapped light within the radiation continuum. *Nature* **499**, 188 (2013).

[3] Zhen, B., Hsu, C. W., Lu, L., Stone, A. D. & Soljačić, M. Topological nature of optical bound states in the continuum. *Physical Review Letters* **113**, 257401 (2014).

[4] Synge, J. L. Flux of energy for elastic waves in anisotropic media. In *Proceedings of the Royal Irish Academy. Section A: Mathematical and Physical Sciences*, vol. 58, 13–21 (JSTOR, 1956).

[5] Koshelev, K., Lepeshov, S., Liu, M., Bogdanov, A. & Kivshar, Y. Asymmetric metasurfaces with high-q resonances governed by bound states in the continuum. *Physical Review Letters* **121**, 193903 (2018).

[6] Purcell, E. Spontaneous emission probabilities at radio frequencies. *Physical Review* **69**, 681 (1946).

[7] Regan, E. C., Igarashi, Y., Zhen, B., Kaminer, I., Hsu, C. W., Shen, Y., Joannopoulos, J. D. & Soljačić, M. Direct imaging of isofrequency contours in photonic structures. *Science Advances* **2**, e1601591 (2016).

[8] Chan, J. *Laser cooling of an optomechanical crystal resonator to its quantum ground state of motion*. Ph.D. thesis, California Institute of Technology (2012).

[9] MacCabe, G. S., Ren, H., Luo, J., Cohen, J. D., Zhou, H., Sipahigil, A., Mirhosseini, M. & Painter, O. Phononic bandgap nano-acoustic cavity with ultralong phonon lifetime. *arXiv preprint arXiv:1901.04129* (2019).

[10] Nowick, A. S. & Berry, B. S. *Anelastic relaxation in crystalline solids* (Academic Press, 1972).

[11] Kleiman, R., Agnolet, G. & Bishop, D. Two-level systems observed in the mechanical properties of single-crystal silicon at low temperatures. *Physical Review Letters* **59**, 2079 (1987).

[12] Phillips, W. Two-level states in glasses. *Reports on Progress in Physics* **50**, 1657 (1987).
